# Phosphate of Lime in Scrofula and Other Depraved States of the System*Some notice of this article having appeared in the newspapers, we have been repeatedly inquired of respecting the phosphate of lime as a remedy in phthisis. We quote the article under the impression that some of our readers may also have been questioned on the subject.— *Editor Buffalo Medical Journal*.

**Published:** 1851-12

**Authors:** 


* *Phosphate of Lime in Scrofula and other depraved states of the System.* By W. Stone, M. D., Prof. of Surgery in the University of Louisiana.

In the July number of the reprint of the London Lancet, there is an article by Beneke, entitled the Physiology and Pathology of the Oxalate and Phosphate of Lime, and their relation to the formation of cells. The conclusions of the author are based upon careful chemical research, and results from the use of the remedy. His researches show, that in man, as well as in vegetables and inferior animals, phosphate of lime as well as albumen and fat, is absolutely essential for the formation of cells, and he considers that many of the pathological states of the system depend upon a deficiency of this salt. The affections in which it is advised, are ulcerations dependent upon a general dyscrasia, and not a mere local affection; infantile atrophy; in those suffering from rickets and consequent diarrhœa and tuberculous diseases, particularly of the lungs in the early stages. I was favorably impressed with the article, and being encouraged by the results of the practice, I am

* Some notice of this article having appeared in the newspapers, we have been repeatedly inquired of respecting the phosphate of lime as a remedy in phthisis. We quote the article under the impression that some of our readers may also have been questioned on the subject.— *Editor Buffalo Medical Journal.*

induced to relate a few cases by way of calling the attention of the profession to it, believing great improvement may be made in the treatment of diseases dependent upon vice of nutrition.

Case I. Slave Bob was admitted into my Infirmary, early in July, with a disease of his nose. Two large fungous growths, one on each side of the nose, barely separated by a strip of sound skin in the centre, of about one inch in diameter, extended nearly to the corners of the eyes. The cavities of the nose were filled by a similar growth, and the disease was making its appearance in the roof of the mouth. His general appearance was bad, and not unlike that of a dirt eater. He complained of pains in different parts of the body, but not much at the seat of the disease, and he had an indolent swelling on one of his feet which finally softened down, and on being opened, discharged a thin matter and broken down tissue, leaving an ill-conditioned ulcer. I had to rely upon him for his history, which must necessarily be imperfect. He said the disease commenced four months previous to the nasal cavities and gradually made its way through. An examination showed that the bones had been absorbed — the mass bled freely, and, upon pressure, a thick, cream-like pus appeared, and some of it resembled softened tuberculous matter. Pulse feeble and frequent, and digestion bad. I do not know what particular treatment he had been under, but he appeared to be slightly under the influence of mercury, and I put him upon the use of the hydriod of potass — cut off the fungus externally, and extracted as much as was practicable from the nasal cavities with polypus forceps, and used a lotion of the sulphate of copper. No perceptible improvement followed, and on the first of August I put him upon the use of cod-liver oil, but his digestion continued bad, had acid eructations, which he thought was worse when he took the oil. The phosphate of lime was added, eight grains three times a day, and he soon began, for the first time, to improve. His color began to return — the local disease began to assume a better appearance. Local treatment was disregarded, and the oil and phosphate of lime has been continued up to this time. His color is now of a shining, healthy black The fungus is even with the surrounding skin. Cicatrization is taking place, and the fungus has disappeared from the nasal cavities, so that he breathes quite freely through them. Those having confidence in cod-liver oil, may attribute the favorable change to it alone, but I would say that no favorable change took place until the lime was given, although it had been given sufficiently, I think, for a fair trial. The oil may supply one deficiency, and the lime another; but my object is not to theorize, but to draw attention. Bleeding, leeching, cups, and gum water, on the one hand, and tonics, stimulants, and opium on the other, are sufficiently well understood, but I believe that chemistry is yet to assist us, and enable us to relieve many of those undefinable maladies that depend upon vices of nutrition, either hereditary or acquired, which cut off so many before the natural decay of the system takes place.

Case II. Miss ——, aged 24, had been in delicate health for some time, without suffering from any particular disease. In May last, a dry cough commenced, and loss of appetite followed, etc. But, to make it brief, as it is but a common case, I saw her about the middle of June, and found the upper part of both lungs filled with tubercles, in some places beginning to soften. Her cough was almost incessant, expectoration slight, consisting of viscid mucus, streaked with pus, and occasionally with blood; pulse a hundred and twenty, much emaciated, and her menses had ceased. She had fever in the evening, and exhausting night sweats. I ordered cod-liver oil, together with a soothing cough mixture, for temporary relief, and to procure rest, which she could not get without. This course afforded some relief, but the appetite did not improve, and I could not say that any marked improvement had taken place. About the first of July I gave the phosphate of lime, in addition to the oil, and in a short time there appeared to be some improvement in the appetite; the sweats began to leave, and her color gradually to return. The same course has been continued up to the present time, and she says she feels better than she has for two years. Her cough is in a great manner gone; she has gained considerable flesh, and has, for the last two periods, menstruated more naturally than for two years previously. There could be no doubt as to the precise nature of this case, and I am free to allow full credit to the oil, but I am confident that the lime was equally useful. The patient, who knows nothing of the medicine, spoke of its good effects. If the theory upon which its beneficial effects are based, is correct, it ought to be an admirable assistant to the oil. I do not pretend that this patient is effectually cured, but it must be admitted that the result of the treatment is highly encouraging. It was a case of unmixed phthisis, that might have been expected to terminate in the course of a few months.

Case III. A child of Mr. W., aged about seven years, had been laboring under a derangement of his bowels something over a year, and had been treated by very excellent physicians, with only temporary benefit. I saw him first in July, during one of his bad spells, as it was termed. I will not be so tedious as to give all the symptoms of the case, or the treatment that had been pursued. Suffice it to say, that he was emaciated very much, but there was no evidence of any serious organic lesion, and no decided appearance of a scrofulous taint. Dyspeptic diarrhœa is a term as applicable as any one term, to his case, though at times he seemed to digest tolerably well; but there was no assimilation or appropriation of his food. He was at first put upon the use of hydriodate of potash, in an infusion of gentian, without any change, and finding that he suffered from acidity, and added the phosphate of lime in doses of six or eight grains, three times a day, which he is still using. I saw him a few days since, and learned from the parents that he had had no new attack, and that his bowels had been steadily improving, and is gaining flesh and strength rapidly. The parents, who are highly intelligent, attribute most of the benefit to the phosphate of lime.— *N. O. Medical Register.*

